# Clinimetric properties of the ASAS health index in a cohort of Italian patients with axial spondyloarthritis

**DOI:** 10.1186/s12955-016-0463-1

**Published:** 2016-05-17

**Authors:** Marco Di Carlo, Valentina Lato, Marina Carotti, Fausto Salaffi

**Affiliations:** Rheumatology Department, Polytechnic University of the Marche, Jesi Ancona, Italy; Radiology Department, Polytechnic University of the Marche, Ancona, Italy

**Keywords:** ASAS HI, Axial spondyloarthritis, Health-related quality of life, Feasibility, Reliability, Validity

## Abstract

**Background:**

The impact of axial spondyloarthritis (axSpA) is considerable in many aspects of the life. Over the last decades, many efforts have been conducted to develop useful tools for the evaluation of disease activity. However, since the development of Assessment of SpondyloArthritis international Society Health Index (ASAS HI), no specific freely questionnaire to describe the overall picture of impairments, limitations and restrictions in activities or social partecipation were available. The aims of this study were to test the feasibility, reliability, and construct validity of the ASAS HI, in order to compare its clinimetric properties with the current available measures of disease activity, functional limitation and health status assessments in patients with axSpA.

**Methods:**

A cohort of 140 consecutive axSpA has been the object of study. The feasibility has been determined by the percentage of patients who were able to complete the questionnaire by themselves and by the time employed to fill the ASAS HI. The reliability has been evaluated performing a test-retest of the questionnaire within a week. The construct validity was examined in three ways. First, we examined construct convergent validity by correlating the scores of the ASAS HI with the Ankylosing Spondylitis Disease Activity Score (ASDAS)-CRP/ESR, the Simplified Ankylosing Spondylitis Disease Activity Score (SASDAS), the Bath Ankylosing Spondylitis Disease Activity Index (BASDAI), the Bath Ankylosing Spondylitis Metrology Index (BASMI), the Bath Ankylosing Spondylitis Functional Index (BASFI), the Ankylosing Spondylitis Quality of Life scale (ASQoL) and the EuroQoL Five Dimensional Questionnaire (EQ-5D). Secondly, we have created patient groups based on the patients' activity ranks (ASDAS-CRP and SASDAS categorisation) within the cohort to assess discriminative accuracy. Additionally, to distinguish patients with active and non-active disease and to assess their respective cut-off points values, the receiver operating characteristic (ROC) curve analysis was used. Thirdly, we analyzed the contribution of demographic (age, sex, and disease duration) and clinical variables (number of comorbidity and disease activity by ASAS-CRP) to the attainment of an ASAS HI condition by stepwise logistic regression.

**Results:**

The mean time to complete the ASAS HI was 1.92 ± 0.76 min. Overall, the ASAS HI questionnaire was correctly completed by the majority of the patients (99,2 %). Coefficients of agreement between ASAS HI scores on first and second administrations were excellent and all items showed very good agreement (ICC = 0.976; range 0.966 to 0.982). The ASAS HI was correlated significantly with all other comparator scores (*p* <0.0001). The highest correlations were seen with ASQoL (rho 0.784; *p* <0.0001), BASFI (rho 0.671; *p* <0.0001) and SASDAS (rho 0.640; *p* <0.0003). On categorizing patients into different cut-off point of disease activity, with respect to the both ASDAS-CRP and SASDAS, ASAS HI scores were highly significantly different between the four categories (*p* <0.0001). An ASAS HI value of 4.0 resulted the cut-off with the highest combination of sensitivity and specificity (82.6 % and 86.3 %, respectively) to define the inactive disease. In the logistic regression model, high disease activity measured by ASDAS-CRP (coefficient 2.39; *p* <0.0001), was the only independent variable associated with ASAS HI.

**Conclusions:**

The results reported in this study confirm the feasibility, reliability and validity of the ASAS HI in Italian patients with axSpA. Even if ASAS HI is not a disease activity index, of particular interest appears the cut-off value of 4.0, under which could be defined the inactive disease. This value could represent an easily applicable starting point in daily clinical practice.

**Electronic supplementary material:**

The online version of this article (doi:10.1186/s12955-016-0463-1) contains supplementary material, which is available to authorized users.

## Background

Axial spondyloarthritis (axSpA) include diseases with predominantly axial involvement, such as ankylosing spondylitis (AS), psoriatic arthritis (PsA) and non-radiographic axial SpA (nr-axSpA) which have as key symptoms both inflammatory back pain and stiffness [1–3].

The impact of AS and nr-axSpa in many aspects of life is considerable, not only about pain, fatigue, stiffness, limitation in activities and in social partecipation, but also in terms of concern about the appeareance, the future and the medication side effects [4, 5].

Over the last decade, significant progresses have been achieved in the development and validation of new tools for the evaluation of disease activity in axSpA [6, 7]. Most of them are based on self-reported questionnaires, such as the Bath Ankylosing Spondylitis Disease Activity Index (BASDAI) [8], which is the more frequently used in clinical trials, or the AS Disease Activity Score (ASDAS) [9], recently proposed by a working Group of the Assessment of SpondyloArthritis international Society (ASAS) for the evaluation of disease activity in patients with AS. ASDAS is the first validated disease activity system that combines both patient-reported outcome (PRO) measures and acute-phase reactants levels. Sommerfleck et al. developed a simplified version of the ASDAS, named Simplified Ankylosing Spondylitis Disease Activity Score (SASDAS) [10]. This score, keeping the sensitive characteristics of the ASDAS, can be considered an intuitive and easy way to assess the disease activity in patients with axSpA.

Thus, while in axSpA are disposable instruments to measure disease activity, but also radiographic damage [11], and magnetic resonance imaging (MRI) inflammation [12], no specific freely available questionnaires to describe the overall picture of impairments, limitations and restrictions in activities or social partecipation were at hand since the creation of ASAS Health Index (ASAS HI) [13]. This tool has been recently developed by Kiltz et al. [13, 14] to assess health in patients with AS according to the International Classification of Functioning, Disability and Health (ICF) categories [15]. The 17 statements of ASAS HI have been obtained from a pool of 251 items originating from questionnaires already in use for patients with axSpA or from questionnaires linked to the ICF. These statements address to the ICF categories of pain, emotional functions, sleep, sexual function, mobility, self-care and community life. So ASAS HI can provide information about the whole range of common difficulties experienced by patients with axSpA [13]. The ASAS HI has been translated into 15 languages, including the Italian.

Taking into account these informations we addressed the aims of our study in the following points: to test the feasibility, reliability, and construct validity of the ASAS HI in order to define its clinimetric properties and to compare its discriminant validity with the current available measures of disease activity, functional limitation and health status assessments in patients with axSpA.

## Methods

### Patient characteristics

From May 2015 to October 2015 have been enrolled 140 consecutive axial SpA patients. The classification of axSpA was based on fulfillment of the ASAS classification criteria that are defined as follows: the presence of sacroiliitis by radiography or by MRI plus at least one SpA feature (“imaging arm”) or the presence of HLA-B27 plus at least two SpA features (“clinical arm”) [2, 3]. Exclusion criteria were the following: other active concomitant musculoskeletal diseases (e.g. gout, calcium pyrophosphate dyhidrate crystal deposition, rheumatoid arthritis), history of cancer or lymphoproliferative disease, uncontrolled diabetes, unstable ischemic heart disease, congestive heart failure, active inflammatory bowel disease, positive serology for hepatitis B, history of active tuberculosis and concomitant fibromyalgia. All patients were treated with non-steroidal antiinflammatory drugs on an on-demand basis. A total 99 patients were on TNF-blockers (70.7 %), including infliximab (39 patients), adalimumab (27 patients), etanercept (22 patients), golimumab (nine patients) and certolizumab pegol (two patients). The choice of the TNF blocking agent was based on the judgment of the rheumatologist and/or on the specific needs of the patient. Patients were allowed to receive concomitant medications as usual in daily clinical practice. All patients were attending the outpatient and inpatient clinics of the Rheumatology Department of the Polytechnic University of the Marche (Jesi, Ancona, Italy) and they represent a “real life” sample of axSpA. The study was approved by the Hospital Clinic ethics committee. All patients agreed to be enrolled in the study and signed informed consent. During the routine visits to our Clinic, a comprehensive questionnaire package has been administered to the patients. The package included the socio-demographic data (sex, age, disease duration and years of school attendance), the ASAS HI [13], the Bath Ankylosing Spondylitis Disease Activity Index (BASDAI) [8], the Bath Ankylosing Spondylitis Functional Index (BASFI) [16], the EuroQoL Five Dimensional Questionnaire (EQ-5D) [17], and the Ankylosing Spondylitis Quality of Life Scale (ASQoL) [18]. The presence of the following co-morbidities was also assessed: hypertension, myocardial infarction, lower extremity arterial disease, major neurological problems, diabetes, gastrointestinal disease, chronic respiratory disease, kidney disease, and poor vision. The ESR (mm/hour) and CRP serum levels (mg/dl) were also collected. All patients gave their informed verbal consent for anonymous analysis of data.

### ASAS HI questionnaire

The ASAS HI questionnaire is composed by 17 items, expressed in the the first person and in present tense, with dichotomous response option: “I agree” and “I do not agree”. Each positive answer is scored 1 while a negative answer is scored 0. The final result is the sum of individual items. Higher values reflect a major degree of impairments, limitations and restrictions. For ASAS HI questionnaire has been performed a re-test: each patient has been invited to fill in for a second time the ASAS HI at home within 1 week from the first administration. This re-test has been handed over during the consecutive visit to our Clinic. In our cohort of patients has been used the online available ASAS HI Italian translation [19].

### Other functional limitation and health status assessments

The Bath Ankylosing Spondylitis Metrology Index (BASMI) quantifies the mobility of the axial skeleton in AS patients and allow objective assessment of clinically significant changes in spinal movement. It includes clinical measures of cervical rotation, tragus to wall distance, lumbar flexion, lumbar side flexion, and intermalleolar distance. Each item is scored from 0 to 10 based on individually defined cut-points. Ranges are given as cervical rotation (>85.0° to <8.6°), tragus to wall distance (<10 cm to >36 cm), lumbar flexion (>7.0 cm to <0.8 cm), lumbar side flexion (>20.0 cm to <1.2 cm), and intermalleolar distance (>119 cm to <30 cm) [20]. Individual scores are averaged to give a final score between 0 and 10, where a higher value reflects more significant impairment of spinal mobility.

The BASFI is composed by ten questions elaborated to determine the degree of functional limitation in patients with AS. Each question is answered using an 11-points numerical rating scale (NRS), with a recall period of the past week. The mean of the ten scales affords the BASFI score – a value between 0 and 10, with a lower score indicating less functional limitation [16]. In this study the paper formats previously validated in the Italian language of the BASFI and the BASDAI have been employed [21].

The ASQoL measure the impact of AS on health-related quality of life (HRQoL) from the patient’s perspective [18]. The questionnaire includes items related to the impact of disease on sleep, mood, motivation, coping, activities of daily living, independence, relationships, and social life. Dichotomous responses, with 0 scored for a “no” and 1 scored for a “yes” for each item. Total score is the sum of the individual responses. Score range is 0–18, with higher scores reflecting greater impairment of HRQoL.

The EQ-5D health state classifier consists of five single-item dimensions - mobility, self-care, usual activities, pain/discomfort, and anxiety/depression - with three levels of response for no, some, or extreme problems in each dimension [17]. In addition to the health state classifier, patients rated their current health on a 20-cm visual analog scale (EQ-5D VAS) ranging from 0 (worst possible health state) to 100 (best possible health state). Responses to these five dimensions are converted into one of 243 different EQ-5D health state descriptions, which range between no problems on all five dimensions (11111) and severe/extreme problems on all five dimensions (33333). The Italian population-based values were used to convert patient responses to the health state classifier into a single index, which produces scores from 1 to − 0.38 [22].

### Measures of disease activity

The BASDAI has six 11-points NRS to measure the severity of fatigue, spinal and peripheral joint pain, localised tenderness, and morning stiffness in patients with AS. Each item is provided using a 0–10 horizontal NRS, to extremes the adjectival descriptors “none” and “very severe”. Item six (duration of morning stiffness) is related to a time scale (0–2 h). The mean of items five (severity of morning stiffness) and six is calculated separately. The BASDAI, a number from 0 to 10, is obtained with the mean of this result with the previous four items. Lower score are indicating lower disease activity. The cut-off of four is used to define the presence of an active disease [8].

The ASDAS is a composite index to measure disease activity in AS, including both self-reported items and objective measures [9]. The score includes assessment of back pain (question 2 of BASDAI), duration of morning stiffness (question 6 of BASDAI), peripheral joint pain and/or swelling (question 3 of BASDAI), PaGA, and a serologic marker of inflammation (ESR or CPR). Five items are combined to give a single disease activity score. The ASDAS has been validated and found to be discriminatory in assessing disease activity in axSpA and it has been endorsed by the ASAS and by the Outcome Measures in Rheumatology (OMERACT). The published cut-offs of ASDAS are the following: <1.3 for inactive disease, ≥1.3 and <2.1 for moderate disease activity, ≥2.1 and <3.5 for high disease activity and ≥3.5 for very high disease activity. A gain ≥1.1 units is considered as a “clinically important improvement”, while an amelioration ≥2.0 represents a “major improvement” [23, 24].

The SASDAS is a simplified version of ASDAS developed by Sommerfleck et al. and can be considered an intuitive and easy way to assess the disease activity in patients with AS [10]. While the equation used to calculate the ASDAS is relatively complex (since requires a calculator), SASDAS can be quickly assessed in the busy daily clinical practice showing a similar good discriminative ability and correlation with different constructs of disease activity and health status compared to the ASDAS [25].

### Statistical analysis

Continuous data were presented as means with standard deviations (SDs) or medians with interquartile range) depending on the distribution of the data (tested with the Kolmogorov–Smirnov test). Categorical data were presented as proportions. Demographic and clinical measures were compared using Mann–Whitney “U” test and chi-square analysis for discontinuous variables. To check for significant systematic differences in test-retest administration, intraclass correlation coefficients (ICC) with 95 % confidence intervals (95 % CI) for mean values were employed. ICC >0.75 were considered relevant [26]. The operational qualities or the feasibility of ASAS HI questionnaires were investigated by the percentage of patients who were able to complete the questionnaire by themselves and by the time employed in filling it out. Within a 1-week interval, patients were asked by the same data collector to repeat ASAS HI, without having access to any previous ratings. Considering the possibility of a change in the patient’s condition over a 1-week interval, a global rating-of-change questionnaire was concurrently administered to the subjects. This so-called “transition questionnaire” investigated the patient’s current health status compared with that when the first questionnaire was completed (question: “compared to when you completed the questionnaire regarding your health status a week ago, how is your health now?”). Possible response options were “much better”, “slightly better”, “no change,” “slightly worse,” or “much worse”. Subjects who reported no change were considered stable and those who reported a change were eliminated from this analysis. In this study, test-retest reliability was analyzed in a group of 125 patients who reported no change in their health. Agreement between scores was also illustrated by Bland and Altman plots, in which the difference between scores was plotted on the y-axis against the average of scores on the x-axis. The construct validity of the ASAS HI was examined in three ways. First, we examined construct convergent validity by correlating the scores of the ASAS HI with ASDAS-CRP/ESR, SASDAS, BASDAI, BASMI, BASFI, ASQoL and EQ-5D. A specific subscale is expected to converge with the scores of those instruments targeting the same construct and to deviate from the scores given by instruments or scales assessing a different one (divergent validity). Spearman’s rho correlation coefficients were obtained to quantify these relationships. Correlations >0.90 were interpreted as very high, 0.70–0.89 as high, 0.50–0.69 as moderate, 0.26–0.49 as low and ≤0.25 as little if any correlation occurred. Secondly, we explored the discriminative accuracy of the ASAS HI questionnaire. For this purpose, we have created patient groups based on the patients’ activity ranks within the cohort and used Kruskal-Wallis test for continuous variables to assess differences. The ASDAS-CRP scores were categorised into four groups: <1.3 for inactive disease, ≥1.3 and <2.1 for moderate disease activity, ≥2.1 and <3.5 for high disease activity, and ≥3.5 for very high disease activity [23]. Similarly, the SASDAS scores were categorised into four groups as follows: inactive disease from 0 to 7.8 (inactive disease), from 7.9 to 13.8 (moderate disease activity), from 13.9 to 27.6 (high disease activity), and above 27.6 (very high activity) [10]. Additionally, to distinguish patients with active and non-active disease and to assess their respective cut off points values, the receiver operating characteristic (ROC) curve analysis was used. The criteria for inactive disease for ASDAS and SASDAS were applied as external criteria. ROC curves were created by plotting the true-positive proportion (sensitivity) versus the false-positive proportion (100-specificity) for the discrimination between inactive and active patients for multiple cut-off points. The area under the ROC curve (AUC) was calculated to quantify the discriminative accuracy. According to Swets [27], AUC from 0.50 to about 0.70 represent poor accuracy, those from 0.70 to 0.90 are “useful for some purposes”, and higher values represent high accuracy. From the ROC curves, we computed the optimal cut off point corresponding to the maximum sum of sensitivity and specificity. The non-parametric Wilcoxon’s signed ranks test is used for calculation and comparison of the areas under the ROC curves, as suggested by Hanley and McNeil [28]. Finally, we analyzed the contribution of demographic (age, sex, and disease duration) and clinical variables (number of comorbidity and disease activity by ASAS-CRP) to the attainment of the ASAS HI condition by stepwise logistic regression. Others clinical variables such as BASMI, BASFI, BASDAI, SASDAS, ASQoL and EQ-5D were excluded for collinearity. All data were entered into a Microsoft Excel database which was developed for the management of the cross-sectional study. All the statistical analyses were performed using the MedCalc® version 11.5 (MedCalc Software, Mariakerke, Belgium).

## Results

### Cohort distribution

The mean age of the 140 patients examined was 46.2 ± 12.0 years (range 22–77). One hundred and one of the respondents were male (72.1 %), 39 were female (27.9 %). The mean duration of disease was 6.7 ± 4.8 years. Ninety-eight patients (70.0 %) had AS while 42 (30.0 %) had nr-axSpA. The mean value (SD) of ASDAS-CRP was 2.2 (0.9), in the range of high disease activity, as long as the mean (SD) BASDAI resulted 3.5 (1.8). The mean values (SD) of BASMI, BASFI and ASQoL were respectively 3.1 (2.1), 3.6 (2.4) and 7.9 (5.1) (see Additional file 1). The 48.5 % had receveid a primary school education and 19.5 % had receveid a high school education. At baseline, 111 patients (79.3 %) had one or more concurrent diseases. The most commonly reported diseases were hypertension (71 patients, 50.7 %) and metabolic disorders (40 patients, 28.6 %). Regarding the extra-articular manifestations, 15 patients (10.7 %) had a simultaneous inflammatory bowel disease, respectively ten patients (7.1 %) Crohn disease and five patients (3.6 %) ulcerative colitis, while 11 patients (7.9 %) had at least one episode of anterior uveitis.

### Score distributions of the ASAS HI

**Fig. 1:**
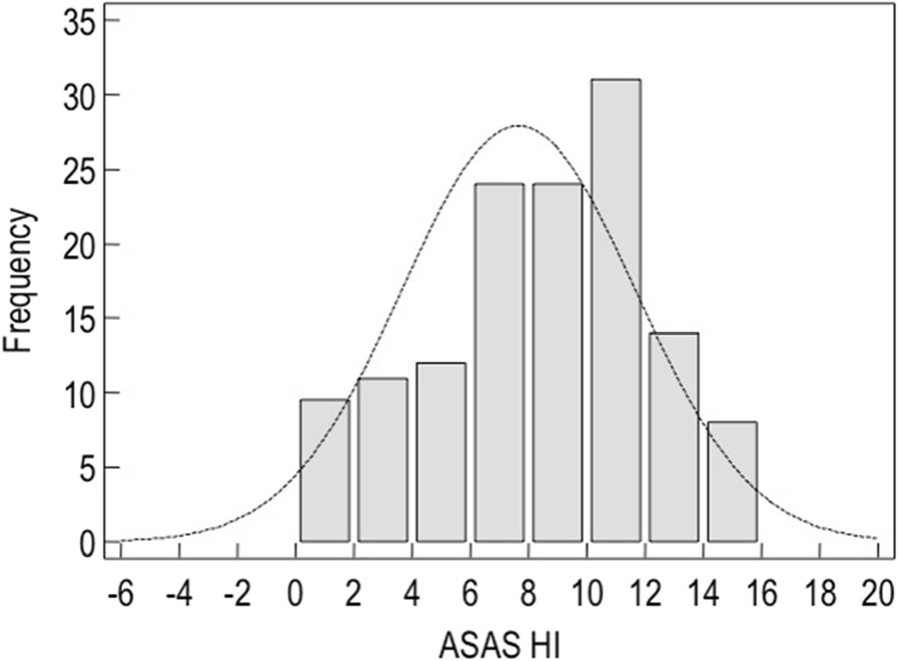
Histograms demonstrating the range and the distribution of ASAS HI questionnaires values. The horizontal axis shows the scores (range 0–17), with high scores indicating a worse health status

Figure 1 presents estimates of central tendency and distribution of score for ASAS HI in all patients at baseline (*n* = 140 patients). The bar on the left of each graph represents the number of subjects with a score of 0 (floor effect); the bar on the right represents the number of subjects with a maximum possible score (ceiling effect). ASAS HI showed a non-normal distribution. The mean (SD) was 7.6 (3.9) and the median (IQR) was 8.0 (4.5–11.0) (see Additional file 2).

### Test-retest reliability and feasibility

**Fig. 2:**
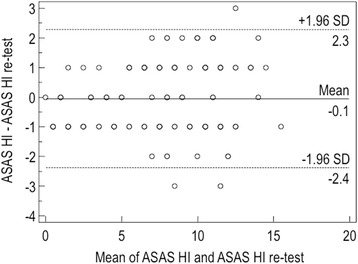
Bland-Altman plot of repeatibility with the differences in ASAS HI values plotted against average values. Ninety-seven percent of the differences against the means were less than two standars deviations (SD; *dotted lines*)

Mean time between the questionnaire administrations was 5.8 days (range 4–7 days). Coefficients of agreement between ASAS HI scores on first and second administrations were excellent. All items showed very good agreement (ICC = 0.976; range 0.966 to 0.982). According to Bland and Altman analysis, there was no systematic error in ASAS HI and in scores (Fig. 2). The mean time to complete the ASAS HI questionnaire by patients was 1.92 ± 0.76 min (range 0.8–3.5 min). Overall, the ASAS HI questionnaire was correctly completed by most respondents (99.2 %). Less than 1 % of each of the ASAS HI questions had missing values. Only four patients (2.9 %) refused to answer to the statement number 7 (interest in sex).

### Concurrent validity

**Fig. 3:**
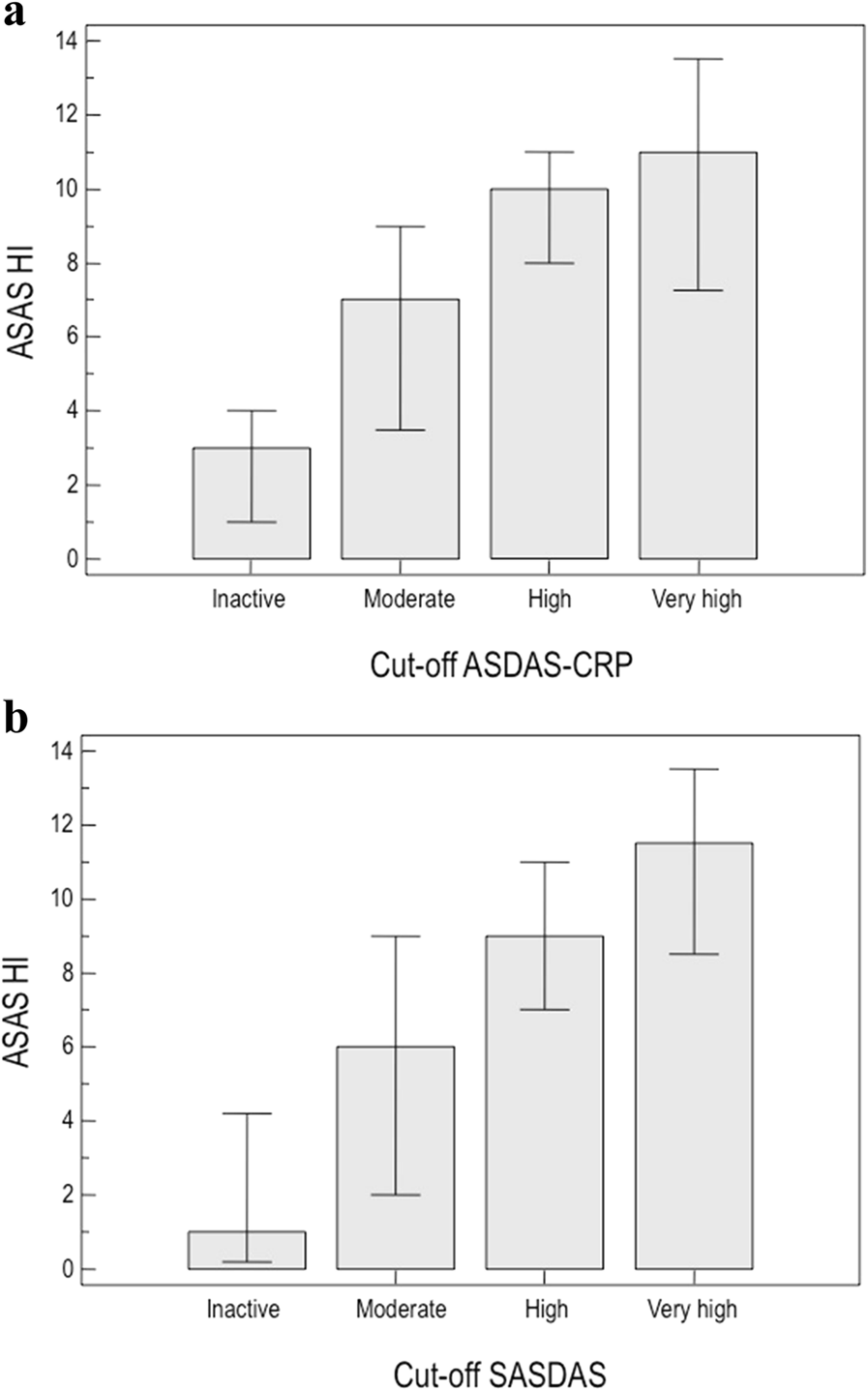
Distribution of ASAS HI scores in relation to different ASDAS-CRP (**a**) and SASDAS (**b**) cut-off of disease activity. The figure shows the mean values of ASDAS HI on the basis of disease activity cut-off points of ASDAS-CRP (**a**) and SASDAS (**b**). The Kruskal-Wallis test was carried out across all four groups (*p* <0.0001)

The ASAS HI was correlated significantly with all other comparator scores (*p* <0.0001) (Table 1). The highest correlations were seen with ASQoL (rho 0.784; *p* <0.0001), BASFI (rho 0.671; *p* <0.0001) and SASDAS (rho 0.640; *p* <0.0003). Strong correlations were also found with ASDAS-ESR (rho 0.580; *p* <0.0001), ASDAS-CRP (rho 0.564; *p* <0.0001), BASDAI (rho 0.568; *p* <0.0001) and EQ-5D (rho 0.460; *p* <0.0001). Moderate correlation was found between ASAS HI and BASMI (rho 0.303; *p* = 0.0001) (Table 1). With respect to the age and disease duration, ASAS HI did not show any significant correlation. Categorizing patients according to the proposed ASDAS-CRP and SASDAS disease activity scoring system revealed 23 patients (16.43 %) with inactive disease, 36 patients (25.71 %) with moderate disease activity, 70 patients (50.00 %) with high disease activity, and 11 patients (7.86 %) with very high disease activity for ASDAS-CRP; 25 patients (17.86 %) with inactive disease, 20 patients (14.29 %) with moderate disease activity, 83 patients (59.29 %) with high disease activity, and 12 patients (8.56 %) with very high disease activity for SASDAS. The cross-classification showed a significant agreement (weighted Kappa 0.79 with standard error of 0.048). On categorizing patients into these different cut-off point of disease activity, with respect to the both ASDAS-CRP and SASDAS, ASAS HI scores were highly significantly different between the four categories (*p* <0.0001) (Fig. 3a and b) (Kruskal-Wallis test).

**Table 1:** Convergent validity analysis: correlation matrix (Spearmanʼs rho) of the ASAS HI Questionnaire versus anthropometric measures (BASMI), specific and generic HRQoL questionnaires (ASQoL and EQ-5D), functional disability (BASFI) and disease activity scores (BASDAI, ASDAS-CRP and SASDAS)

	ASQoL	BASDAI	BASFI	BASMI	EQ-5D	SASDAS	ASDAS-CRP
ASAS HI	0.784	0.568	0.671	0.303	−0.460	0.640	0.564
	<0.0001	<0.0001	<0.0001	0.0003	<0.0001	<0.0001	<0.0001
ASQoL		0.645	0.626	0.271	−0.436	0.689	0.620
		<0.0001	<0.0001	0.0012	<0.0001	<0.0001	<0.0001
BASDAI			0.586	0.192	−0.453	0.868	0.757
			<0.0001	0.0229	<0.0001	<0.0001	<0.0001
BASFI				0.578	−0.329	0.723	0.624
				<0.0001	0.0001	<0.0001	<0.0001
BASMI					−0.160	0.321	0.289
					0.0590	0.0001	0.0005
EQ-5D						−0.407	−0.418
						<0.0001	<0.0001
SASDAS							0.845
							<0.0001

Spearman rank correlation coefficient

*Abbreviations*: *ASAS HI* Assessment of SpondyloArthritis international Society Health Index, *ASQoL* Ankylosing Spondylitis Quality of Life Scale, *BASDAI* Bath Ankylosing Spondylitis Disease Activity Index, *BASFI* Bath Ankylosing Spondylitis Functional Index, *BASMI* Bath Ankylosing Spondylitis Metrology Index,*EQ-5D* EuroQoL Five Dimensional Questionnaire, *SASDAS* Simplified Ankylosing Spondylitis Disease Activity Index, *ASDAS* Ankylosing Spondylitis Disease Activity Score, *CRP* C-Reactive Protein

### Discriminant validity

**Fig. 4:**
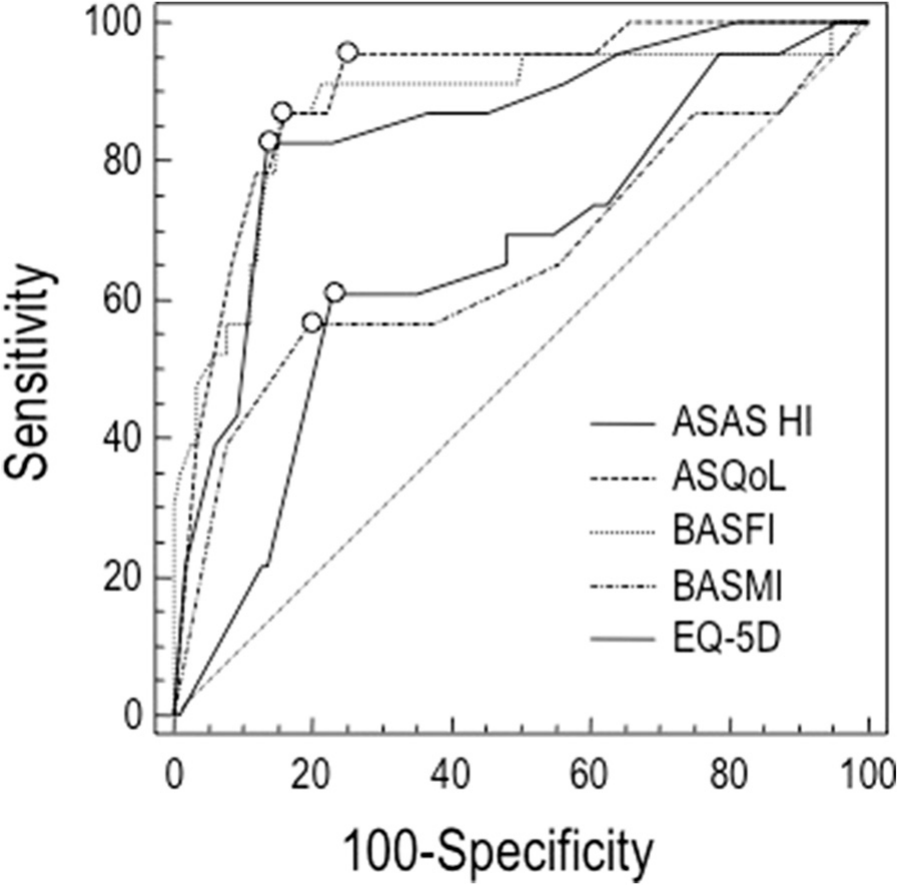
The ROC curves to discriminate the ability of ASAS HI to distinguish patients with active and inactive disease in comparison with anthropometric measures and self-report questionnaires, using ASDAS-CRP as external indicator. The area under the ROC curve (AUC) in this setting can be interpreted as the probability of correctly identifying the improved patients from non-improved. A line that runs diagonally across the figure from lower left to upper right will have an area of 0.5; this represents an instrument that does not discriminate

The ROC curves to discriminate the ability of ASAS HI to distinguish patients with active and inactive disease were similar to ASQoL and BASFI (Table 2 and Fig. 4). The discriminatory power of ASAS HI was very good, without significant difference, with an AUC of 0.850 (95 % CI 0.763 ± 0.938) (differences between areas of the ASQol = 0.053 ± 0.038 with 95 % C.I. from 0.022 to 0.128; *p* = 0.1691 and differences between areas of the BASFI = 0.029 ± 0.045 with 95 % C.I. from 0.060 to 0.118; *p* = 0.521). From these data, we calculated the cut-off values of ASAS HI for inactive disease with the highest combination of sensitivity and specificity. The resulting cut-off value was 4.0 (sensitivity 82.6 %; specificity 86.3 %) with a LR+ of 6.04 (see Additional file 3).

**Table 2:** AUC-ROC curve values (standard error and 95 % confidence intervals) to distinguish patients with active and non-active disease, were similar for ASAS HI, ASQoL and BASFI questionnaires

	AUC	SE^a^	95 % CI^b^
ASAS HI	0.850	0.044	0.763 to 0.938
ASQoL	0.903	0.032	0.840 to 0.967
BASFI	0.880	0.046	0.790 to 0.969
BASMI	0.657	0.073	0.515 to 0.800
EQ-5D	0.656	0.062	0.534 to 0.778

For abbreviations see Table 1

^a^Hanley & McNeil, 1982

^b^AUC ± 1.96 SE

### Variables associated with ASAS HI

In the logistic regression model (Table 3), high disease activity measured by ASDAS-CRP (coefficient 2.39; *p* <0.0001) was the only independent variable associated with ASAS HI. Other predictors such as age, sex, disease duration, educational level and comorbidity were not clinically important contributors to questionnaire.

**Table 3:** Summary of the results of regression analyses, with regression coefficients for the predictor variable

Independent variables	Coefficient	Std. Error	r_partial_	t	P
(Constant)	1.7544				
ASDAS-CRP	2.3956	0.3291	0.5338	7.280	<0.0001
Disease duration	−0.0764	0.0421	−0.1555	−1.815	0.0718
Educational level	−0.0574	0.0823	−0.0604	−0.697	0.4868
Gender	0.0125	0.6898	0.0016	0.0181	0.9856
Age, years	0.0209	0.0255	0.0709	0.819	0.4142
Comorbidity	0.2910	0.1546	0.1611	1.883	0.0619

For abbreviations see Table 1

## Discussion

The severity of axSpA is defined by different aspects of the disease, such as disease activity, damage, reduced motility, reduced physical function, reduced social partecipation and work ability, with considerable costs, frequently hidden and not easy to quantify [4, 5, 29].

Moreover, patients with problems due to extra-articular organ involvement related to axSpa such as uveitis, inflammatory bowel disease, and psoriasis, undergo to additional disadvantages.

Data from clinical trials and observational cohorts have shown that the burden of the disease in nr-axSpA is comparable to that in patients with AS [30, 31]. In our study we considered both patients affected by AS and nr-axSpA.

A precise meaning of the severity of axSpA has to include several categories. The core set of “what to measure”, because it is typical and relevant for functioning and health in patients with AS, has been established in 2010 by the ASAS on the basis of the ICF, published by World Health Organition (WHO) [32]. ICF is a universally agreed and understood framework to define the spectrum of problem in functioning. This classification endorses the bio-psyco-social model, recognising the importance of environmental and personal factors on functionig and disability [15]. In the context of the ICF, the term “functioning” belongs to a concept of health and global functioning, wider than the physical function. In clinical setting ICF is used not only for a functional status assessment, but also for goal setting, treatment planning and monitoring, as well as outcome measurement.

ASAS HI has been developed with the purpose to measure the overall picture of impairments, limitations and restrictions due to AS.

Thus, while previous instruments have been focused on specific symptoms, physical function and HRQoL [16, 18], ASAS HI is the first PRO disease-specific, based on the categories of ASAS/WHO comprehensive ICF core set for AS [13]. The use of the ASAS HI seems feasible in clinical practice, since it contains only 17 dichotomous items.

As described by our results, it is also a reliable tool, with a strong agreement beetween two test within a week.

Even if ASAS HI is a health index and not a HRQoL instrument, the highest correlation in terms of concurrent validity was found with ASQoL, one of the most relevant questionnaire in the evaluation of QoL in patients with AS. A good concurrent validity has been also found in comparison with the other indices, except BASMI, that is an anthropometric measure [20].

Among the proposed indices to evaluate disease activity, BASDAI is the most frequently used in clinical trials and in daily practice as yet. However BASDAI could be and ambiguos measure of disease activity that does not capture the entire spectrum of problems [33].

Respect to BASDAI, the ASDAS (or the simplified SASDAS) is an index that reflects several aspects of disease activity and correlates well with both physician’s and patient’s perception of disease activity.

ASAS HI is not a measure of disease activity, notwithstanding our data showed the capability of the instrument to categorize the patients into different cut-off point of disease activity, with respect to the both ASDAS and SASDAS. Of particular interest appears the cut-off value of 4.0, under this value patients could be defined to have inactive disease. This value could represent an easily applicable starting point in daily clinical practice in patients with axSpA.

Moreover, for its features, we think that ASAS HI could be usefully adaptable for electronic systems capturing PROs [34], and this topic will be object of future researchs.

The major limitations to this study are represented by specific restrictions of each analytic method. A primary limitation which must be emphasized is that responsiveness of ASAS HI has not been studied over a long period of time. A further potential limitation that has to be considered regarding the presented results is due to a non-randomly selected primary care sample.

## Conclusions

The results reported in this study confirm the feasibility, reliability and validity of the Italian version of ASAS HI in patients with axSpA. Although we have not yet studied the sensitivity of ASAS HI to change (i.e., responsiveness), this study has some implications for the conduction of future clinical trials in axSpA. Its generalisability and usefulness in assessing treatment and long-term outcomes now need to be evaluated in broader settings. Such kind of analyses are currently underway.

## Additional files

Additional file 1: Descriptive statistics of disease activity scores (BASDAI, ASDAS-CRP and SASDAS), anthropometric measures (BASMI), functional disability index (BASFI) and specific HRQoL questionnaire (ASQoL). (DOC 55 kb) Additional file 2: Descriptive statistics of the ASAS HI. (DOC 33 kb) Additional file 3: Area under the ROC curve (AUC) of the ASAS HI, criterion values and coordinates of the ROC curve. (DOC 50 kb)
