# Supplementary material for: Clinimetric properties of the ASAS health index in a cohort of Italian patients with axial spondyloarthritis
Source: Health Qual Life Outcomes. 2016 May 17;14:78. doi: 10.1186/s12955-016-0463-1 (PMC4869300; doi:10.1186/s12955-016-0463-1)
Supplement: Additional file 1: — Descriptive statistics of disease activity scores (BASDAI, ASDAS-CRP and SASDAS), anthropometric measures (BASMI), functional disability index (BASFI) and specific HRQoL questionnaire (ASQoL). (DOC 55 kb) [file 12955_2016_463_MOESM1_ESM.doc]

**Descriptive statistics of disease activity scores (BASDAI, ASDAS-CRP and SASDAS), anthropometric measures (BASMI), functional disability index (BASFI) and specific HRQoL questionnaire (ASQoL)**

|  | **BASDAI** | **ASDAS-CRP** | **SASDAS** |
| --- | --- | --- | --- |
| Sample size | 140 | 140 | 140 |
| Lowest value | [0.0000](cmd:SHOWXMINMAX?56) | [0.5000](cmd:SHOWXMINMAX?48) | [2.1000](cmd:SHOWXMINMAX?48) |
| Highest value | [9.0000](cmd:SHOWXMINMAX?38) | [4.8000](cmd:SHOWXMINMAX?38) | [35.3000](cmd:SHOWXMINMAX?27) |
| Arithmetic mean | 3.5482 | 2.2295 | 16.8336 |
| 95% CI for the mean | 3.2398 to 3.8566 | 2.0819 to 2.3771 | 15.4910 to 18.1761 |
| Median | 3.7000 | 2.2190 | 17.3000 |
| 95% CI for the median | 3.3750 to 4.0000 | 2.0975 to 2.4596 | 15.4924 to 19.2000 |
| Variance | 3.4066 | 0.7806 | 64.5512 |
| Standard deviation | 1.8457 | 0.8835 | 8.0344 |
| Relative standard deviation | 0.5202 (52.02%) | 0.3963 (39.63%) | 0.4773 (47.73%) |
| Standard error of the mean | 0.1560 | 0.07467 | 0.6790 |
| Coefficient of Skewness | -0.08205 (P=0.6816) | 0.2143 (P=0.2877) | -0.03314 (P=0.8683) |
| Coefficient of Kurtosis | -0.3308 (P=0.4095) | -0.1154 (P=0.8862) | -0.5722 (P=0.0704) |
| Kolmogorov-Smirnov test for Normal distribution | accept Normality (P=0.3524) | accept Normality (P=0.8974) | accept Normality (P=0.5209) |

|  | **BASMI** | **BASFI** | **ASQoL** |
| --- | --- | --- | --- |
| Sample size | 140 | 140 | 140 |
| Lowest value | [0.0000](cmd:SHOWXMINMAX?1) | [0.0000](cmd:SHOWXMINMAX?21) | [0.0000](cmd:SHOWXMINMAX?19) |
| Highest value | [9.0000](cmd:SHOWXMINMAX?38) | [8.9000](cmd:SHOWXMINMAX?4) | [17.0000](cmd:SHOWXMINMAX?17) |
| Arithmetic mean | 3.1000 | 3.6396 | 7.8643 |
| 95% CI for the mean | 2.7469 to 3.4531 | 3.2436 to 4.0357 | 7.0274 to 8.7012 |
| Median | 3.0000 | 3.4500 | 8.0000 |
| 95% CI for the median | 3.0000 to 4.0000 | 3.1092 to 3.9908 | 7.0000 to 9.0000 |
| Variance | 4.4647 | 5.6170 | 25.0822 |
| Standard deviation | 2.1130 | 2.3700 | 5.0082 |
| Relative standard deviation | 0.6816 (68.16%) | 0.6512 (65.12%) | 0.6368 (63.68%) |
| Standard error of the mean | 0.1786 | 0.2003 | 0.4233 |
| Coefficient of Skewness | 0.4191 (P=0.0426) | 0.2884 (P=0.1556) | -0.07897 (P=0.6929) |
| Coefficient of Kurtosis | -0.2366 (P=0.6110) | -0.6852 (P=0.0169) | -1.0830 (P<0.0001) |
| Kolmogorov-Smirnov test for Normal distribution | accept Normality (P=0.0779 | accept Normality (P=0.6351) | accept Normality (P=0.0721) |
