# Supplementary material for: Clinimetric properties of the ASAS health index in a cohort of Italian patients with axial spondyloarthritis
Source: Health Qual Life Outcomes. 2016 May 17;14:78. doi: 10.1186/s12955-016-0463-1 (PMC4869300; doi:10.1186/s12955-016-0463-1)
Supplement: Additional file 2: — Descriptive statistics of the ASAS HI. (DOC 33 kb) [file 12955_2016_463_MOESM2_ESM.doc]

**Descriptive statistics of the ASAS HI**

| Lowest value | 0.00 |
| --- | --- |
| Highest value | [15.00](cmd:SHOWXMINMAX?17) |
| Arithmetic mean | 7.66 |
| 95 % CI for the mean | 6.99 to 8.32 |
| Median | 8.00 |
| 95 % CI for the median | 7.00 to 9.00 |
| Variance | 15.99 |
| Standard deviation | 3.99 |
| Relative standard deviation | 0.52 (52.23 %) |
| Standard error of the mean | 0.34 |
| Coefficient of Skewness | -0.35 (P = 0.0829) |
| Coefficient of Kurtosis | -0.80 (P = 0.0018) |
| Kolmogorov-Smirnov test for Normal distribution | accept Normality (P = 0.0625) |
