# Supplementary material for: Clinimetric properties of the ASAS health index in a cohort of Italian patients with axial spondyloarthritis
Source: Health Qual Life Outcomes. 2016 May 17;14:78. doi: 10.1186/s12955-016-0463-1 (PMC4869300; doi:10.1186/s12955-016-0463-1)
Supplement: Additional file 3: — Area under the ROC curve (AUC) of the ASAS HI, criterion values and coordinates of the ROC curve. (DOC 50 kb) [file 12955_2016_463_MOESM3_ESM.doc]

**Area under the ROC curve (AUC) of the ASAS HI, criterion values and coordinates of the ROC curve**

| Area under the ROC curve (AUC) | 0.850 |
| --- | --- |
| Standard Error^a^ | 0.0445 |
| 95 % Confidence interval^b^ | 0.763 to 0.938 |
| z statistic | 7.868 |
| Significance level P (Area = 0.5) | <0.0001 |

^a^ Hanley & McNeil, 1982

^b^ AUC ± 1.96 SE

**Criterion values and coordinates of the ROC curve** [[Show]](javascript:showdiv('d4','d5','table1');)

| Criterion | Sensitivity | 95 % CI | Specificity | 95 % CI | +LR | 95 % CI | -LR | 95 % CI |
| --- | --- | --- | --- | --- | --- | --- | --- | --- |
| <0 | 0.00 | 0.0 - 14.8 | 100.00 | 96.9 - 100.0 |  |  | 1.00 | 1.0 - 1.0 |
| ≤0 | 21.74 | 7.5 - 43.7 | 98.29 | 94.0 - 99.8 | 12.72 | 2.6 - 61.6 | 0.80 | 0.6 - 1.0 |
| ≤1 | 39.13 | 19.7 - 61.5 | 94.02 | 88.1 - 97.6 | 6.54 | 2.7 - 15.8 | 0.65 | 0.5 - 0.9 |
| ≤2 | 43.48 | 23.2 - 65.5 | 90.60 | 83.8 - 95.2 | 4.62 | 2.2 - 9.6 | 0.62 | 0.4 - 0.9 |
| ≤3 | 60.87 | 38.5 - 80.3 | 88.89 | 81.7 - 93.9 | 5.48 | 3.0 - 10.1 | 0.44 | 0.3 - 0.7 |
| **≤4** | **82.61** | **61.2 - 95.0** | **86.32** | **78.7 - 92.0** | **6.04** | **3.7 - 9.9** | **0.20** | **0.08 - 0.5** |
| ≤5 | 82.61 | 61.2 - 95.0 | 82.91 | 74.8 - 89.2 | 4.83 | 3.1 - 7.5 | 0.21 | 0.09 - 0.5 |
| ≤6 | 82.61 | 61.2 - 95.0 | 76.92 | 68.2 - 84.2 | 3.58 | 2.4 - 5.2 | 0.23 | 0.09 - 0.6 |
| ≤7 | 86.96 | 66.4 - 97.2 | 63.25 | 53.8 - 72.0 | 2.37 | 1.8 - 3.1 | 0.21 | 0.07 - 0.6 |
| ≤8 | 86.96 | 66.4 - 97.2 | 54.70 | 45.2 - 63.9 | 1.92 | 1.5 - 2.5 | 0.24 | 0.08 - 0.7 |
| ≤9 | 91.30 | 72.0 - 98.9 | 43.59 | 34.4 - 53.1 | 1.62 | 1.3 - 2.0 | 0.20 | 0.05 - 0.8 |
| ≤10 | 95.65 | 78.1 - 99.9 | 35.90 | 27.2 - 45.3 | 1.49 | 1.3 - 1.8 | 0.12 | 0.02 - 0.8 |
| ≤11 | 100.00 | 85.2 - 100.0 | 18.80 | 12.2 - 27.1 | 1.23 | 1.1 - 1.3 | 0.00 |  |
| ≤12 | 100.00 | 85.2 - 100.0 | 8.55 | 4.2 - 15.2 | 1.09 | 1.0 - 1.2 | 0.00 |  |
| ≤13 | 100.00 | 85.2 - 100.0 | 6.84 | 3.0 - 13.0 | 1.07 | 1.0 - 1.1 | 0.00 |  |
| ≤14 | 100.00 | 85.2 - 100.0 | 2.56 | 0.5 - 7.3 | 1.03 | 1.0 - 1.1 | 0.00 |  |
| ≤15 | 100.00 | 85.2 - 100.0 | 0.00 | 0.0 - 3.1 | 1.00 | 1.0 - 1.0 |  |  |
